# LncRNA NBR2 Inhibits the Malignancy of Thyroid Cancer, Associated With Enhancing the AMPK Signaling

**DOI:** 10.3389/fonc.2020.00956

**Published:** 2020-06-12

**Authors:** Wen Yang, Zhikun Zheng, Pengfei Yi, Shi Wang, Ning Zhang, Jie Ming, Jie Tan, Hui Guo

**Affiliations:** ^1^Department of Breast and Thyroid Surgery, Union Hospital, Tongji Medical College, Huazhong University of Science and Technology, Wuhan, China; ^2^Department of Thoracic Surgery, Union Hospital, Tongji Medical College, Huazhong University of Science and Technology, Wuhan, China

**Keywords:** LncRNA NBR2, thyroid cancer, malignancy, AMPK, EMT

## Abstract

Long non-coding RNA NBR2 is a transcript of the neighbor of BRCA1 gene 2 and can regulate tumor development. However, there is little information on the role of NBR2 in the progression of thyroid cancers (TC). Here, we show that NBR2 expression is down-regulated in TC tissues and associated with histologic subtypes of TC. NBR2 expression was variably reduced in different TC cells. While NBR2 silencing significantly enhanced the malignancy of BCPAP cells by increasing cell proliferation, clonogenicity, wound healing, and invasion as well as tumor growth *in vivo*, and decreasing spontaneous apoptosis, NBR2 over-expression had opposite effects in BHT101 cells. Furthermore, treatment with A-769662 (a specific AMPK activator), like NBR2 over-expression, significantly attenuated the malignancy of BHT101 cells while treatment with Compound C (a specific AMPK inhibitor) significantly rescued that NBR2-reduced malignancy of BHT101 cells. In comparison with non-tumor thyroid epithelial Nthy-ori 3-1 cells, obviously increased GLUT-1 expression, but decreased AMPK and ACC phosphorylation were detected in TC cells. While NBR2 silencing further enhanced GLUT-1 expression and reduced AMPK and ACC phosphorylation as well as the EMT process in BCPAP cells. NBR2 over-expression also had opposite effects in BHT101 cells. Similar patterns of GLUT-1 expression and AMPK and ACC phosphorylation were detected in the different types of xenograft TC tumors *in vivo*. Therefore, such data indicated that NBR2 acted as a tumor suppressor of thyroid cancers associated with enhancing the AMPK signaling and NBR2 may be a potential biomarker and therapeutic target for thyroid cancers.

## Introduction

Thyroid cancer (TC) is the most frequent endocrine malignant cancer (1) and its incidence is increasing worldwide (2, 3). TC accounts for about 2% of cancer cases diagnosed newly in the world (4, 5). Pathologically, TCs can be classified into well-differentiated papillary thyroid cancer (PTC) and follicular thyroid cancer (FTC), poorly differentiated thyroid cancer (PDTC), and undifferentiated or anaplastic thyroid cancer (ATC). TC has been thought to be a cumulative mutant tumor (6). Many TCs begin as well-differentiated cancers and progress into PDTC and ATC by their dedifferentiation. However, the mechanisms underlying TC progression is not clear and need to be urgently investigated.

Long non-coding RNAs (lncRNAs) are the type of RNAs with a length of more than 200 nucleotides and usually do not code for a protein (7–9). LncRNAs are important factors participating in the process of many biological functions, including the development and progression of malignant tumors, such as TC (10–12). Previous studies have shown that LncRNA XIST acts as a ceRNA of miR-34a to modulate the cell proliferation and tumor growth of TC by enhancing the MET/PI3K/AKT signaling (13). The LncRNA gene for PTCSC2 is located in rs965513, the most significant known predisposing variant of PTC, in chromosome 9q22 and regulates the carcinogenesis (14). LncRNA HOXA-AS2 facilitates tumorigenesis and progression of PTC by enhancing the Wnt/β-catenin signaling (15) while LncRNA PTCSC3 attenuates drug resistance of ATC to chemotherapeutic drugs by inhibiting the STAT3/INO80 pathway (16). Furthermore, LncRNA SPRY4-IT promotes the progression of TC by targeting the TGF-β/Smad signaling (17). Therefore, identifying and exploring new lncRNAs that regulate the malignancy of TC may uncover potential therapeutic targets for the treatment of TC.

LncRNA NBR2, a transcript of the neighbor of BRCA1 gene 2, is widely expressed in different organs (18–20). Under an energy stress, lncRNA NBR2 has been shown to suppress tumor progression by enhancing the AMP-activated protein kinase (AMPK) activity (18), and reduces the survival of cancer cells by decreasing GLUT-1 expression (19). A recent study has shown that lncRNA NBR2 can inhibit the proliferation, invasion, and migration of osteosarcoma cells by inhibiting the EMT process (20). However, there is no information on the role of LncRNA NBR2 in the malignancy of TC. In this study, we first analyzed LncRNA NBR2 expression in TC from TCGA, and tested the impact of altered LncRNA NBR2 expression on the malignancy of TC and its AMPK activation and GLUT-1 expression *in vitro* and *in vivo*.

## Materials and Methods

### Data Acquisition

The expression of NBR2 and relationship with the clinicopathological features of TC patients were analyzed through UALCAN (http://ualcan.path.uab.edu) and GEPIA (http://gepia.cancer-pku.cn/). The TC patient raw data containing RNA sequencing (RNA seq) and clinical information were obtained from The Cancer Genome Atlas (TCGA) repository website (http://firebrowse.org/).

### Reagents and Antibodies

Compound C (an AMPK inhibitor) and A-769662 (an AMPK activator) were purchased from Selleck. The primary antibodies anti-pAMPK, anti-pACC, anti-GLUT-1, and anti-β-actin were obtained from MDL, anti-E-cadherin, anti-N-cadherin, anti-Vimentin, and anti-cleaved-caspase 3 (Asp175) were purchased from Affinity.

### Cell Culture

Human primary thyroid follicular non-tumor Nthy-ori 3-1 cells and TC BCPAP and BHT101 cells were purchased from the American Type Culture Collection (ATCC, Rockville, MD). Nthy-ori 3-1, BCPAP, and BHT101 cells were cultured in RPMI 1640 or DMEM with 10% fetal bovine serum (FBS) at 37°C in 5% CO_2_. The cells were exposed to fresh medium every 2 days to maintain cell viability.

### Cell Transfection

BCPAP cells were transduced with lentivirus for expression of NRB2-specific shRNA or control scramble RNA (GeneChem, Shanghai, China) at a multiplicity of infection (MOI) of 10. The shRNA was chosen based on preliminary screening of three siRNAs (Guangzhou RiboBio, China) using Lipofectamine 3000 (Invitrogen, USA) (Supplementary Table 1). Furthermore, BHT101 cells were transduced with control lentivirus or lentivirus for expression of NBR2. The efficacy of NRB2 silencing or over-expression was determined by quantitative RT-PCR.

### Quantitative RT-PCR (qRT-PCR)

Total RNA was extracted from cells or tumor tissues by RNAiso Plus (TaKaRa, Kyoto, Japan) and was reversely transcribed to cDNA using the PrimeScript™ RT reagent Kit (TaKaRa). The qRT-PCR was performed using SYBR Master Mix (TaKaRa) and specific primers (Supplementary Table 2) in a Bio-Rad CFX96 system. The relative levels of NBR2 to β-actin transcripts were analyzed by 2^−ΔΔ*Ct*^ method.

### Cell Proliferation Assay

The cell proliferation was measured using Cell Counting Kit-8 Assay (Beyotime, Shanghai, China), according to the manufactures' instruction. Briefly, TC cells (0.5 × 10^4^ cells/well) were cultured in a 96-well-plate in the presence or absence of Compound C (20 μM) or A-769662 (100 μM) and the cell viability was measured longitudinally by detecting absorbance at 450 nm in a microplate reader (BIO-RAD, 170-6750).

### Cell Invasion Assay

The different groups of cells (2 × 10^4^ cells/well) were cultured in FBS-free medium in the presence or absence of Compound C (20 μM) or A-769662 (100 μM) in the top chamber that had been coated with Matrigel (Sigma) in transwell plates (8 μm pore polycarbonate membranes, Corning-Costar, USA). The bottom chambers were filled with complete medium (10% FBS medium). After culture for 24 h, the cells on the upper surface of chamber membrane were removed. The invaded cells on the bottom surface of the chamber membranes were fixed with 4% formaldehyde and stained with crystal violet. The invaded cells were photoimaged and counted in a blinded manner.

### Wound Healing Assay

The different groups of cells were cultured in 12-well-plates and when the cells reached 90–100% of confluence, the monolayer of cells were wounded using a sterile pipette tip. After being washed with PBS, the cells were cultured in FBS-free medium in the presence or absence of Compound C (20 μM) or A-769662 (100 μM) up to 72 h. The cells were photoimaged longitudinally and the wounded areas were measured 0, 24, 48, and 72 h post-wounding.

### Clonogenic Assay

The different groups of cells (500 cells/well) were cultured in 6-well-plates in the presence or absence of Compound C (20 μM) or A-769662 (100 μM) for 7 days. The cell colonies were fixed in 4% paraformaldehyde and stained with crystal violet, followed by photoimaged. The cell colonies were counted in a blinded manner.

### Cell Apoptosis Assay

The different groups of cells in logarithmic phase were treated with, or without, Compound C (20 μM) or A-769662 (100 μM) for 24 h, harvested and stained with FITC-Annexin V/propidium iodide (PI). The percentages of apoptotic cells were analyzed by flow cytometry in a BD FACS Flow Cytometer (BD, USA).

### Western Blot Analysis

The different groups of cells were lyzed in cold RIPA buffer containing PMSF (sigma, USA) and centrifuged. After quantified the protein concentrations using the BCA protein assay kit (MDL, MD913053), the cell lysates (50 μg/lane) were separated by sodium dodecyl sulfate polyacrylamide gel electrophoresis (SDS-PAGE) on 10% gels and transferred to polyvinylidene difluoride (PVDF) membranes. The membrane was blocked using 5% non-fat dry milk in TBST and were incubated at 4°C overnight with primary antibodies diluted 1:1,000. After being washed, the bound antibodies were detected with horseradish peroxidase (HRP)-conjugated secondary antibodies and visualized with BeyoECL in a gel imaging system (Bio-Rad). Similarly, the collected tumor tissues were homogenized and subjected to Western blot.

### Tumor Xenograft in Mice

All animal experiments were approved by the Institutional Animal Care and Treatment Committee of Tongji Medical College of Huazhong University of Science and Technology, China. Individual BALB/c nude mice at 4 weeks of age were injected subcutaneously with individual groups of cells (10^7^ cells/mouse, *n* = 5 per group). Their tumor growth was monitored every three days up to 40 days post-inoculation and the tumor volumes were calculated by the formula 0.5 × width^2^ × length. At the end, their tumors were dissected for further experiments.

### Statistical Analysis

Data are expressed as the mean ± standard deviation (SD). Difference between two groups was tested using two-tailed Student's *T*-test or Mann–Whitney *U*-test using SPSS 16.0 statistical software (IBM, NY). A *p*-value of <0.05 was considered statistically significant.

## Results

### Decreased LncRNA NBR2 Expression in TC Tissues Is Associated With TC Progression

**Figure 1:**
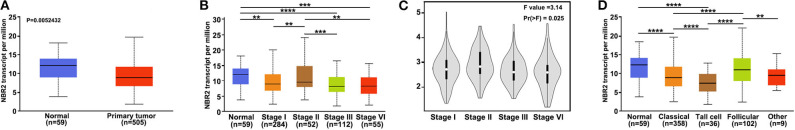
LncRNA NBR2 expression is down-regulated in TC tissues. **(A)** Relative levels of NBR2 transcripts in 505 TC and 59 non-tumor thyroid tissues in TCGA. **(B)** (UALCAN), **(C)** (GEPIA). Stratification analysis of NBR2 expression in the different stages of TC. **(D)** The levels of NBR2 expression in the different histologic subtypes of TC. **P < 0.01; ***P < 0.005; ****P < 0.001.

To explore the role of lncRNA NBR2 in TC, the levels of NBR2 expression in 59 non-tumor thyroid and 505 TC tissues in TCGA database were obtained and analyzed through UALCAN, which was an interactive web-portal to perform in-depth analyses of TCGA gene expression data (21). We found that the NBR2 expression was variable in TC tissues and significantly lower than that in non-tumor thyroid tissues (*P* < 0.05, Figure 1A). Stratification analysis indicated that the levels of NBR2 transcripts were inversely associated with TC stages (Figure 1B) and histologic subtypes (Figure 1D). A similar pattern of NBR2 expression was achieved (*P* < 0.05, Figure 1C) using the GEPIA dataset (http://gepia.cancer-pku.cn/) (22). After normalization with Log_2_, the decreased NRB2 transcripts were significantly associated with histologic subtypes and lower frequency of all types of TC tissues displayed significantly reduced NRB2 transcripts (*P* < 0.05, Table 1). Hence, decreased NRB2 expression was associated with TC progression.

**Table 1:** Association of lncRNA NBR2 expression with clinicopathologic features in TC patients.

**Parameter**	**Total *N* = 500**	**LncRNA NBR2**		**χ^2^**	***P***
		**Low *n* = 125 (%)**	**High *n* = 375 (%)**		
**Age (years)**				2.198	0.138
<55	335	77 (23.0)	258 (77.0)		
≥55	165	48 (29.1)	117 (70.9)		
**Gender**				1.311	0.252
Female	365	96 (26.3)	269 (73.7)		
Male	135	29 (21.5)	107 (79.3)		
**Tumor size**				0.447	0.504
≤ 2 cm	177	41 (23.2)	136 (76.8)		
>2 cm	309	80 (25.9)	229 (74.1)		
**Tumor focality**				0.008	0.927
Unifocal	267	68 (25.5)	199 (74.5)		
Multifocal	227	57 (25.1)	170 (74.9)		
**T classification**				1.345	0.246
T1–T2	307	71 (23.1)	236 (76.9)		
T3–T4	191	53 (27.7)	138 (72.3)		
**Lymph node metastasis**				0.488	0.485
N0	227	56 (24.7)	171 (75.3)		
N1	225	62 (27.6)	163 (72.4)		
**Metastasis**				0.022	0.882
M0	277	70 (25.3)	207 (74.7)		
M1	13	3 (23.1)	10 (76.9)		
**Stage**				1.647	0.199
I–II	334	78 (23.4)	256 (76.6)		
III–IV	164	47 (28.7)	117 (71.3)		
**Histological type (TPC)**				6.278	0.043*
Classical/usual	356	85 (23.9)	271 (76.1)		
Follicular	102	24 (23.5)	78 (76.5)		
Tall cell	35	15 (42.9)	20 (57.1)		

* *P < 0.05*.

### NBR2 Reduces the Malignancy of TC Cells *in vitro*

**Figure 2:**
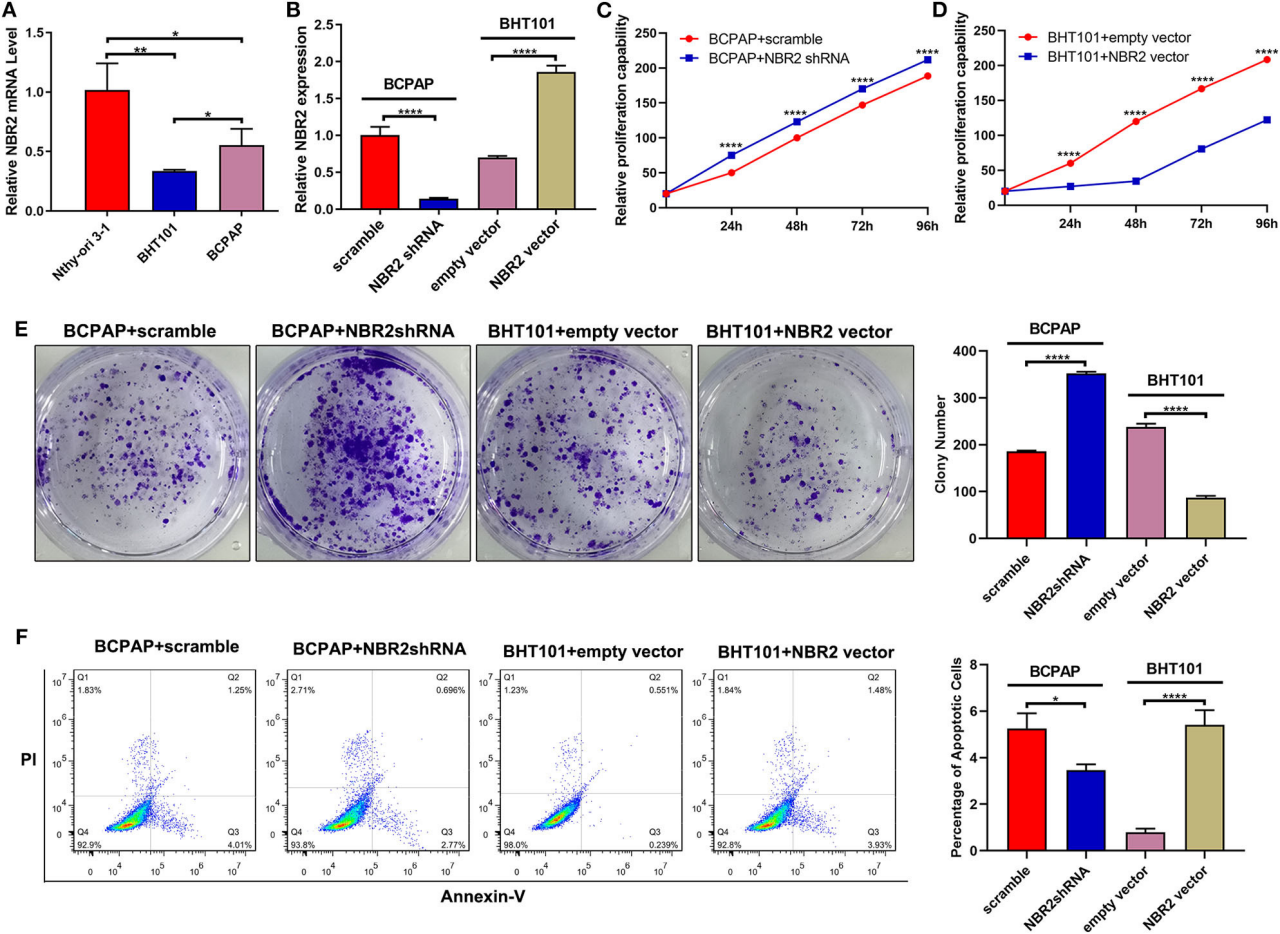
Altered NBR2 expression modulates the proliferation, clonogenicity, and apoptosis of TC cells. **(A)** The NBR2 expression in non-tumor Nthy-ori3-1, TC BCPAP, and BHT101 cells was analyzed by qRT-PCR. **(B)** Altered NBR2 expression was demonstrated in TC cells. **(C,D)** Altered NBR2 expression changed the proliferation of TC cells, determined by CCK-8 assays. **(E)** The colony formation. **(F)** Flow cytometry analysis of apoptotic cells. Data are expressed as the mean ± SD of each group of cells from three separate experiments. *P < 0.05; **P < 0.01; ****P < 0.001.

**Figure 3:**
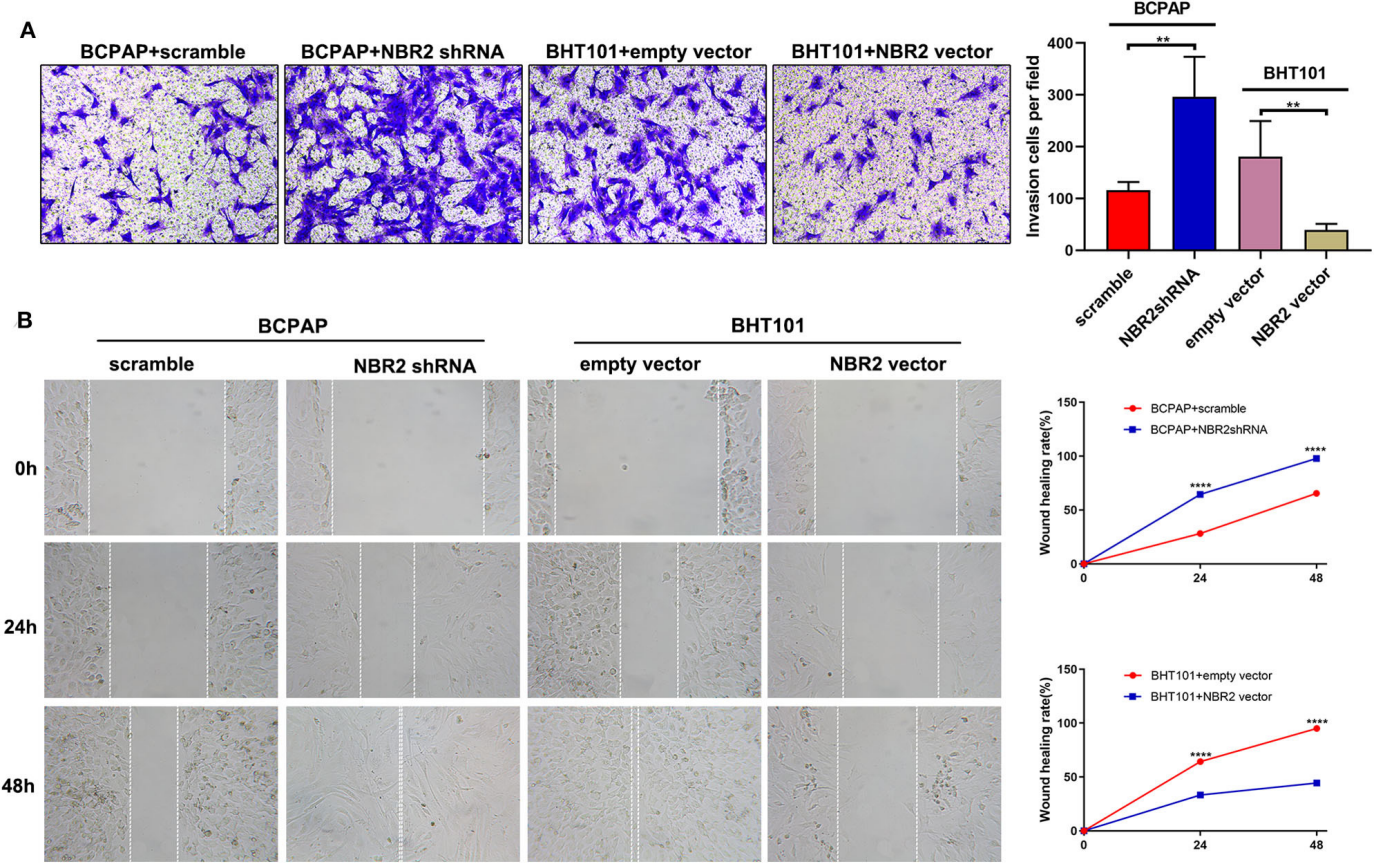
Altered NBR2 expression modulates the invasion and wound healing of TC cells. **(A)** Transwell analysis of cell invasion. **(B)** Wound healing analysis. Data are expressed as the mean ± SD of each group of cells from three separate experiments. **P < 0.01; ****P < 0.001.

**Figure 4:**
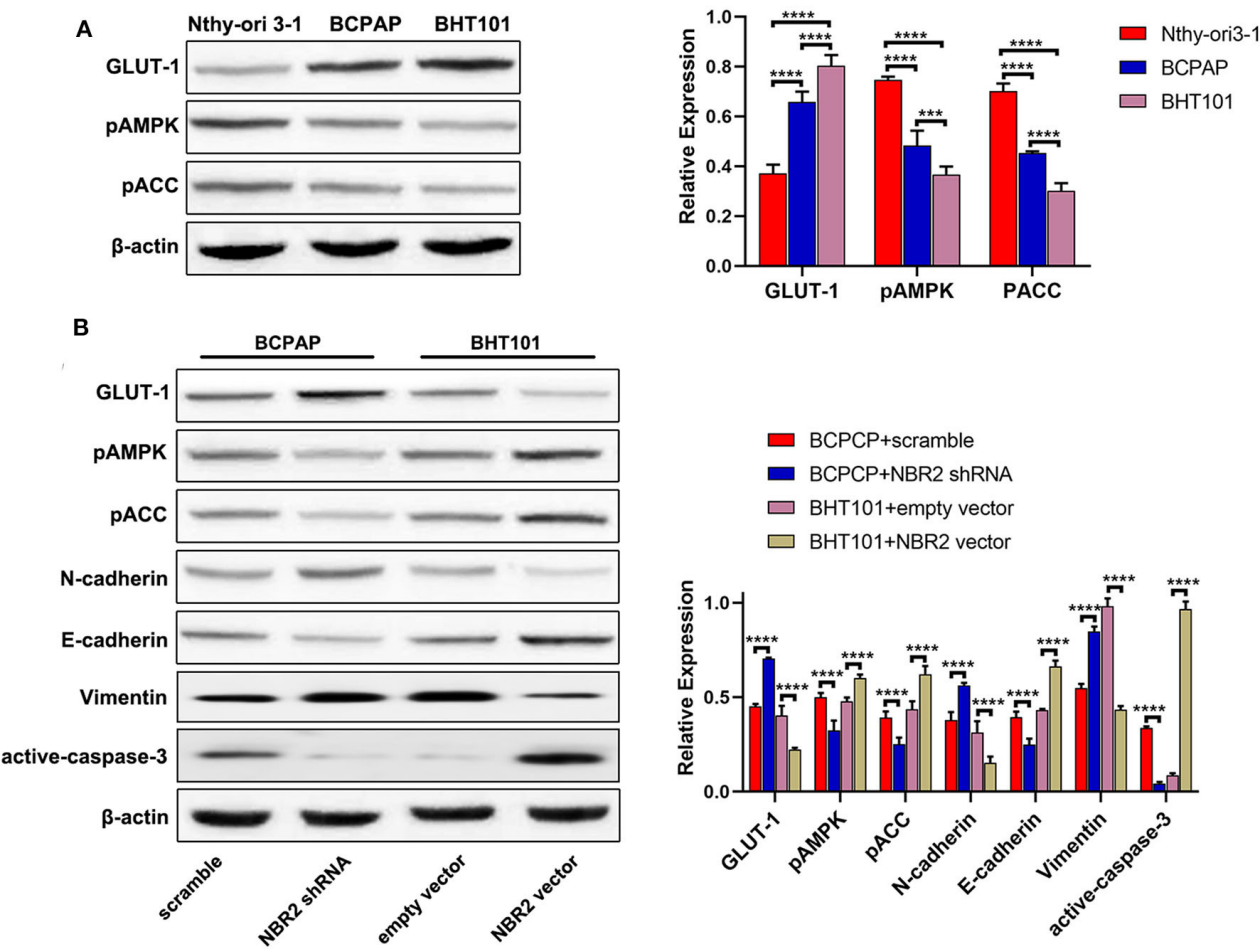
Altered NBR2 expression changes the GLUT-1, E-cadherin, N-cadherin, Vimetin, and cleaved caspase-3 expression and AMPK and ACC phosphorylation in TC cells. **(A)** Western blot analysis of the expression of GLUT-1 expression and AMPK and ACC phosphorylation in Nthy-ori3-1, BCPAP, and BHT101 cells. **(B)** Western blot analysis of the levels of GLUT-1, E-cadherin, N-cadherin, Vimentin, and cleaved-caspase-3 expression and AMPK and ACC phosphorylation in TC cells. Data are representative images and expressed as the mean ± SD of each group of cells from three separate experiments. ***P < 0.005; ****P < 0.001.

Next, we analyzed the levels of NRB2 transcripts in non-tumor thyroid follicular epithelial Nthy-ori 3-1, TC BCPAP, and BHT101 cells and found that the NRB2 transcripts in TC cells were significantly lower than that in Nthy-ori 3-1 cells (Figure 2A). Given that BHT101 cells exhibited the lower NBR2 transcripts than BCPAP cells, we transduced lentivirus for expression of NRB2-specific shRNA (Constructed by valid sequences obtained from screening siRNAs, Supplementary Figure 1) in BCPAP cells and lentivirus for NBR2 over-expression in BHT101 cells, respectively. The efficacy of NBR2 silencing or over-expression was determined by qRT-PCR (Figure 2B). Functionally, NRB2 silencing promoted the malignancy of BCPAP cells by increasing TC cell proliferation and clonogenicity, and decreasing their apoptosis, accompanied by reducing cleaved caspase-3 (Figures 2C,E,F, 4B). In contrast, NRB2 over-expression reduced the malignancy of BHT101 cells by decreasing TC cell proliferation and clonogenicity, and increasing their apoptosis, accompanied by elevating cleaved caspase-3 (Figures 2D–F, 4B). Furthermore, while NRB2 silencing promoted the invasion and wound healing of BCPAP cells and NRB2 over-expression significantly inhibited the invasion and wound healing of BHT101 cells *in vitro* (Figures 3A,B). These opposite effects of altered NRB2 expression indicated that NRB2 attenuated the malignancy of TC cells *in vitro*.

### Altered NBR2 Expression Modulates GLUT-1 Expression, AMPK, and ACC Phosphorylation and EMT in TC Cells

Glucose metabolism and energy regulation as well as the EMT process are crucial for the malignancy of cancers (23, 24). To understand the mechanisms underlying the action of NBR2, we characterized the relative levels of GLUT-1 expression and AMPK and ACC phosphorylation in the different types of cells by Western blot (Figure 4A). In comparison with that in the non-tumor Nthy-ori 3-1 cells, significantly increased GLUT-1 expression, but decreased AMPK and ACC phosphorylation were detected in BCPAP and BHT101 cells. Further Western blot indicated that NBR2 silencing decreased AMPK and ACC phosphorylation and E-cadherin expression, but increased GLUT-1, N-cadherin, and Vimentin expression in BCPAP cells (Figure 4B). In contrast, NBR2 over-expression had opposite effects on BHT101 cells (Figure 4B). Such data indicated that NBR2 expression decreased GLUT-1 expression and the EMT process, but increased AMPK and ACC activation in TC cells *in vitro*.

### Pharmacological Inhibition of AMPK Partially Rescues the NBR2-Decreased Proliferation and Migration of TC Cells

**Figure 5:**
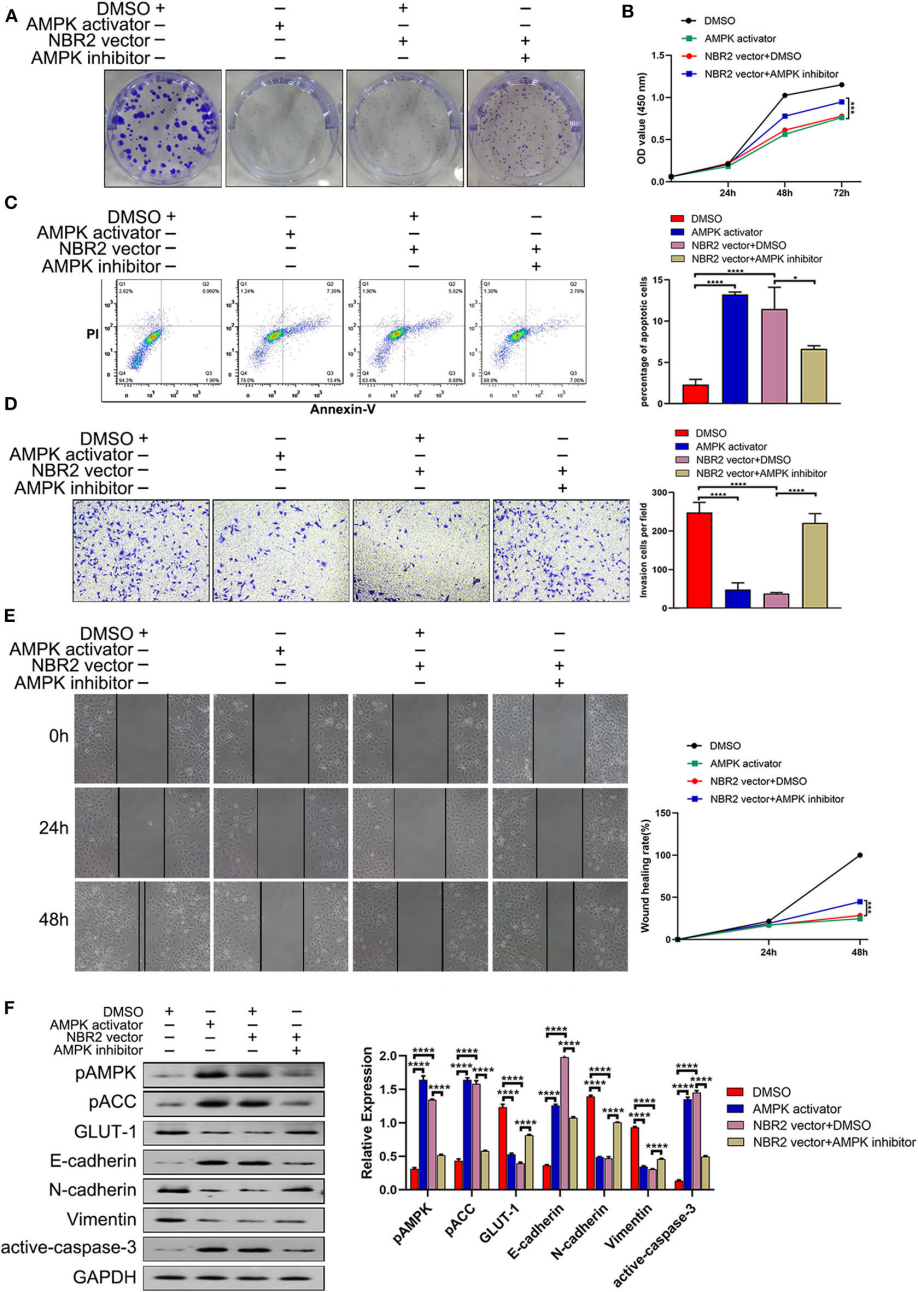
Pharmacological modulation of AMPK activity affects the malignancy of TC cells. BHT101 or NBR2-expressing BHT101 cells were tested for their colony formation, proliferation, apoptosis, invasion, wound healing in the presence or absence of A-769662 (a specific AMPK activator, 100μM) or Compound C (a specific AMPK inhibitor, 20 μM), respectively. Their AMPK/ACC activation, GLUT-1, EMT-related molecule, and cleaved caspase-3 expression were quantified by Western blot assays. **(A)** The colony formation. **(B)** CCK8 analysis of cell proliferation. **(C)** Flow cytometry analysis of apoptotic cells. **(D)** Transwell analysis of cell invasion. **(E)** Wound healing analysis**. (F)** Western blot analysis of the levels of GLUT-1, E-cadherin, N-cadherin, Vimentin, and cleaved-caspase-3 expression and AMPK and ACC phosphorylation in TC cells. Data are representative images or expressed as the mean ± SD of each group of cells from three separate experiments. *P < 0.05; ****P < 0.001.

To understand the importance of AMPK in NBR2-decreased malignancy of TC cells, we tested whether pharmacological modulation of AMPK activity could directly affect or rescue the NBR2-decreased malignancy of TC cells. We found that treatment with 100 μM A-769662, a specific activator of AMPK, significantly decreased the proliferation, clonogenicity, wound healing and invasion of BHT101 cells, accompanied by increased the frequency of apoptotic cells, similar to that of NBR2 over-expression (Figures 5A–E). In contrast, treatment with 20 μM Compound C, a specific inhibitor of AMPK, significantly mitigated the NBR2-decreased proliferation, clonogenicity, would healing, and invasion of BHT101 cells and reduced their apoptosis (Figures 5A–E). Similar to that of NBR2 over-expression, treatment with A-769662 significantly enhanced AMPK and ACC phosphorylation, E-cadherin, and cleaved caspase-3 expression as well as reduced GLUT-1, N-cadherin, and Vimentin expression in BHT101 cells (Figure 5F). In contrast, treatment with Compound C significantly decreased AMPK and ACC phosphorylation, E-cadherin, and cleaved caspase-3 expression, but increased GLUT-1, N-cadherin, and Vimentin expression in the NBR2-over-expressed cells, relative to that of the un-treated NBR2-over-expressed control cells (Figure 5F). Such two independent lines of data demonstrated the importance of AMPK in the NBR2-decreased malignancy of TC.

### NBR2 Inhibits Tumor Growth and AMPK Phosphorylation *in vivo*

**Figure 6:**
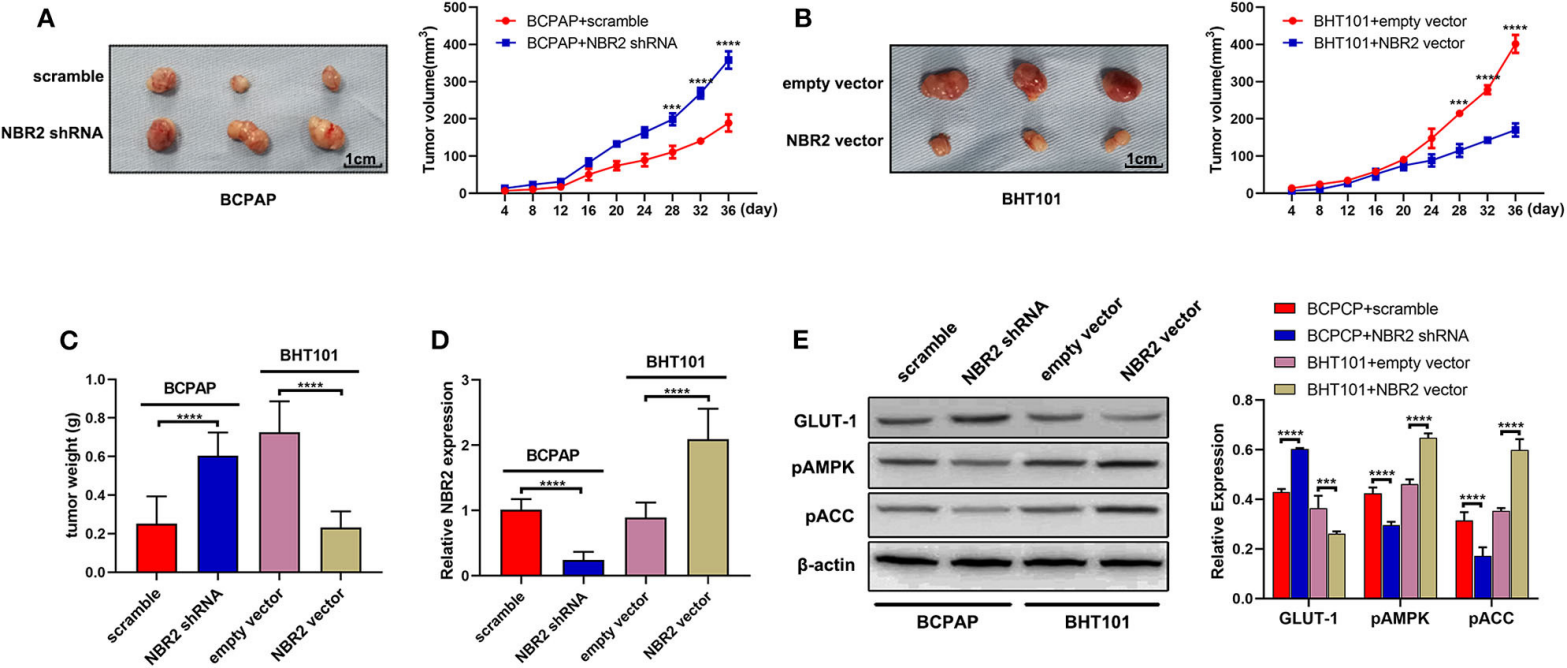
NBR2 inhibits the growth of xenograft TC tumors in mice. **(A,B)** Representative images of tumor xenografts at the end of the experiment (left penal) and the dynamic growth of xenograft tumors (right penal). **(C)** The tumor weights. **(D)** Quantitative RT-PCR analysis of NBR2 expression in tumor tissues. **(E)** Western blot analysis of the levels of GLUT-1 expression, AMPK, and ACC phosphorylation in tumor tissues. Data are representative images or expressed as the mean ± SD of each group of tumors (*n* = 3 per group). ***P < 0.005; ****P < 0.001.

Finally, we tested the role of altered NBR2 expression in xenograft tumors in BALB/c nude mice. As shown in Figures 6A–C, NBR2 silencing significantly increased the growth of BCPAP tumors and their weights while NBR2 over-expression significantly reduced BHT101 tumors and weights in mice. Quantitative RT-PCR analysis revealed the NBR2 expression in the different groups of tumors as expected (Figure 6D). Western blot analysis indicated that NBR2 silencing increased GLUT-1 expression, but decreased AMPK and ACC phosphorylation in BCPAP tumors while NBR2 over-expression had opposite effects (Figure 6E), consistent with our observations *in vitro*. These data demonstrated that NBR2 inhibited the TC growth, associated with its ability to regulate glucose and energy metabolism *in vivo*.

## Discussion

Although most TCs usually have a good prognosis, they can relapse and metastasize into the cervical lymph nodes and distant organs, which are the important factors affecting the life quality. Furthermore, ATC is one of the most malignant TCs with a really poor prognosis, because it can invade and metastasize early. In this study, we found that NBR2 expression was down-regulated in TC tissues and cells, particularly in ATC, extending previous studies in breast and renal cancers (18). Furthermore, NBR2 transcripts were associated with TC stages and histologic subtypes as well as the malignant degrees of TC cells. Such novel data suggest that the levels of NBR2 transcripts may be valuable for classification and prognosis of TC if validated.

A previous study has shown that LncRNA NBR2 inhibits the proliferation, invasion, and migration of osteosarcoma cells (20). We found that lncRNA NBR2 silencing promoted the malignancy of TC cells by enhancing their proliferation, clonogenicity, invasion, and wound healing of TC cells as well as TC growth *in vivo*, but reducing their apoptosis. In contrast, NBR2 over-expression had opposite effects on the malignancy of TC cells. Furthermore, NBR2 silencing increased N-cadherin and Vimentin expression, but decreased E-cadherin expression, the hallmarks of enhanced EMT process in TC cells. In contrast, NBR2 over-expression inhibited the EMT process in TC cells, which may contribute to inhibition of invasion and wound healing in TC cells. These findings indicated that LncRNA NBR2 acted as a tumor suppressor to attenuate the malignancy of TC, similar to observations in osteosarcoma (11). Accordingly, NBR2 may be a new therapeutic target and interventional strategy to increase NBR2 transcripts may be valuable for control of TC.

The AMPK serves as a key sensor of cellular energy status (25), and functionally, the AMPK can switch anabolism into catabolism to restore energy balance in response to energy stress (18). Because many tumors prefer anabolism for their growth, the AMPK activation can inhibit tumor growth (26). Actually, the AMKP activation can attenuate the mTOR signaling and inhibit cancer cell proliferation by phosphorylating the upstream tumor suppressor TSC1 and TSC2 to induce cell cycle arrest through the p53/p21/p27 pathway (25). Conversely, GLUT1 expression is up-regulated in many cancers, and its high expression is often related to a poor prognosis of cancer (27). In this study, we found that the levels of AMPK and ACC phosphorylation decreased in TC cells and were negatively associated with GLUT-1 expression and the malignant degrees of TC cells. Such data extended previous observations that treatment with all-trans retinoic acid (ATRA) induced AMPK activation and decreased GLUT-1 expression (28). Therefore, we speculate that NBR2 can may regulate GLUTI expression and be associated with AMPK activation. Furthermore, NBR2 silencing significantly decreased AMPK and ACC phosphorylation, and increased GLUT-1 expression in TC cells while NBR2 over-expression had opposite effects in TC cells. Similarly, pharmacological activation of AMPK, like NBR2 over-expression, enhanced AMPK and ACC phosphorylation and decreased the malignancy and GLUT-1 expression in TC cells, while inhibition of AMPK partially rescued the NBR2 over-expression-decreased malignancy and GLUT-1 expression and mitigated the NBR2-enhanced AMPK and ACC phosphorylation in TC cells. Such findings strongly suggest that NBR2 may promote the AMPK activation and decrease GLUT-1 expression to attenuate the malignancy of TC cells. We are interested in further investigating the molecular mechanisms underlying the action of NBR2 in regulating the AMPK activation, GLUT-1 expression and energy metabolism in the future studies.

In summary, the results indicated that LncRNA NBR2 expression was down-regulated in TC tissues and associated with TC stages and histologic subtypes as well as inversely correlated with the malignant degrees of TC. Functionally, NBR2 acted as a tumor suppressor to inhibit the malignancy of TC *in vitro* and *in vivo*. Mechanistically, NBR2 promoted the AMPK and ACC activation to down-regulate GLUT-1 expression and the EMT process in TC cells. These novel findings suggest that NBR2 transcripts may be valuable for TC classification and prognosis, and NBR2 may be a new target for design of therapy for control of TC progression.

## Data Availability Statement

The data used to support the findings of this study are available from the corresponding author upon request.

## Ethics Statement

The animal study was reviewed and approved by the Institutional Animal Care and Treatment Committee of Tongji Medical College of Huazhong University of Science and Technology, China.

## Author Contributions

ZZ and HG contributed to the conception and design of the study. PY, SW, and NZ collected and analyzed the data. WY, JM, and JT wrote the manuscript. All authors have read and approved the final manuscript.

## Conflict of Interest

The authors declare that the research was conducted in the absence of any commercial or financial relationships that could be construed as a potential conflict of interest.
