# Corrigendum: LncRNA NBR2 Inhibits the Malignancy of Thyroid Cancer, Associated With Enhancing the AMPK Signaling

**DOI:** 10.3389/fonc.2021.759471

**Published:** 2021-08-27

**Authors:** Wen Yang, Zhikun Zheng, Pengfei Yi, Shi Wang, Ning Zhang, Jie Ming, Jie Tan, Hui Guo

**Affiliations:** ^1^Department of Breast and Thyroid Surgery, Union Hospital, Tongji Medical College, Huazhong University of Science and Technology, Wuhan, China; ^2^Department of Thoracic Surgery, Union Hospital, Tongji Medical College, Huazhong University of Science and Technology, Wuhan, China

**Keywords:** LncRNA NBR2, thyroid cancer, malignancy, AMPK, EMT

In the published article, the co-first authors are three people including the corresponding author Hui Guo, but in fact the co-first authors should be Wen Yang and Zhikun Zheng. Hui Guo is the corresponding author but not the co-first author, so the symbol “†” behind Hui Guo’s name is deleted.

The authors apologize for this error and state that this does not change the scientific conclusions of the article in any way. The original article has been updated.

## Publisher’s Note

All claims expressed in this article are solely those of the authors and do not necessarily represent those of their affiliated organizations, or those of the publisher, the editors and the reviewers. Any product that may be evaluated in this article, or claim that may be made by its manufacturer, is not guaranteed or endorsed by the publisher.

