# Supplementary material for: LncRNA NBR2 Inhibits the Malignancy of Thyroid Cancer, Associated With Enhancing the AMPK Signaling
Source: Front Oncol. 2020 Jun 12;10:956. doi: 10.3389/fonc.2020.00956 (PMC7304297; doi:10.3389/fonc.2020.00956)
Supplement: Supplementary file 1 [file Data_Sheet_1.docx]

Supplementary Table 1. The siRNA sequences

| Sequence(5’-3’） | sense sequences | antisense sequence |
| --- | --- | --- |
| hs-NBR2-si-1 | CAGCGCAAUUGUCAGCUAAdTdT | UUAGCUGACAAUUGCGCUGdTdT |
| hs-NBR2-si-2 | CUGUGUUUGCUGAGGAUAAdTdT | UUAUCCUCAGCAAACACAGdTdT |
| hs-NBR2-si-3 | GAGGCAGGUGGUUCAUCUAdTdT | UAGAUGAACCACCUGCCUCdTdT |

Supplementary Table 2. The primer sequences

| Symbol | primer F | primer R |
| --- | --- | --- |
| actin | GACAGGATGCAGAAGGAGATTACT | TGATCCACATCTGCTGGAAGGT |
| NBR2 | GGAGGTCTCCAGTTTCGGTA | TTGATGTGTGCTTCCTGGG |
