# Supplementary figures and images for: LncRNA NBR2 Inhibits the Malignancy of Thyroid Cancer, Associated With Enhancing the AMPK Signaling
Source: Front Oncol. 2020 Jun 12;10:956. doi: 10.3389/fonc.2020.00956 (PMC7304297; doi:10.3389/fonc.2020.00956)

Supplementary Fig. 1


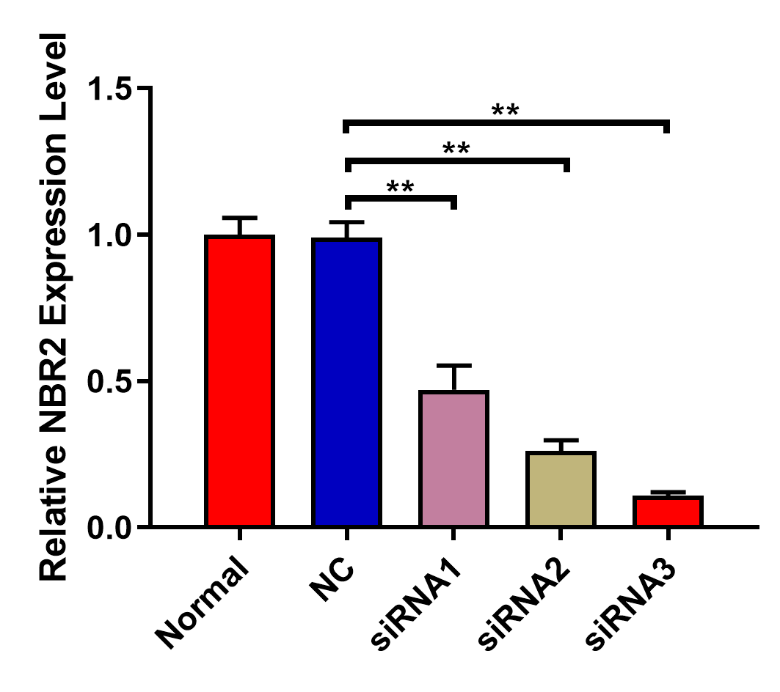


**Figure 1.** Relative NBR2 expression levels of the three siRNA. ** P < 0.01

Supplement: Supplementary file 2 [file Data_Sheet_2.docx]
